# The impact of accent stigma on self-efficacy and acculturation strategy among international students in the United States

**DOI:** 10.3389/fpsyg.2024.1414282

**Published:** 2024-09-05

**Authors:** Xiaodi Yan

**Affiliations:** China University of Political Science and Law, Beijing, China

**Keywords:** accent stigma, self-efficacy, acculturation, integration, international students

## Abstract

**Introduction:**

This paper aimed to understand international students’ experience of accent stigma during interpersonal interactions, and how it affected their self-efficacy and acculturation strategy during intercultural adjustment.

**Methods:**

Study 1 conducted in-depth interviews with 15 international students (60% female, 21.6 years old on average), asking for narratives on how they perceived accent stigma was enacted in everyday scenarios. Study 2 distributed a survey to 132 international students (53.5% female, 25.52 years old on average) with scales measuring perceived accent stigma, perceived discrimination, perceived hate, fear, self-efficacy, and integration into the host culture.

**Results:**

Based on thematic analysis, Study 1 identified verbal disapproval, verbal avoidance, nonverbal disapproval, and nonverbal avoidance as four behavioral markers that signal the enactment of accent stigma during interpersonal interactions. Study 2 assessed a path model and found that accent stigma was associated with more perceived discrimination, perceived hate, and fear. Accent stigma also had negative impacts on self-efficacy, which in turn, resulted in poor integration into the host culture.

**Conclusion:**

This study examined the negative consequences of accent stigma on international students and highlighted the role of self-efficacy during international students’ intercultural adjustment. Findings had important theoretical and practical implications in terms of better supporting and serving international students during their stressful transitional period in a new culture

## 1 Introduction

Over one million international students studied at U.S. higher education institutions during the 2022/23 academic year (IIE Open Doors, 2023). While international students are trying to navigate their way through a new culture, concern with one’s accent present as one of the stressors (Dovchin, 2020; Park et al., 2022; Zhu and Bresnahan, 2021). International students often experience stigma on their non-native accents, which has a series of negative consequences. For instance, international students who are targeted by accent stigma can perceive hostility and hatred from the host culture, and face negative evaluations and discriminatory acts (Dovchin, 2020; McDonough et al., 2022). However, how accent stigma further influenced international students’ intercultural adjustment outcomes were insufficiently explored in exiting literature. Additionally, it is essential to understand the experience of accent stigma from the perceiver’s (i.e., international students) perspective. Therefore, this paper is intended to investigate how accent stigma was perceived by international students, and how perceived accent stigma influenced international students’ intercultural adjustment.

### 1.1 Stigma on non-native accents

Goffman (1963) defined stigma as “a special kind of relationship between attribute and stereotype” (p. 14). Someone with “an attribute that is deeply discrediting” (p. 13) is “a blemished person, …polluted, to be avoided” (p. 11), and is “reduced in our minds from a whole and usual person” (p. 12) and “disqualified from full social acceptance” (p. 11). Stigma occurs when an individual characteristic is humiliating and belittled in a social context (Crocker et al., 1998; Phelan et al., 2008; Major and O’Brien, 2005). Accent is one of such undesirable characteristics that is subject to devaluation, criticism, and discrimination (Dragojevic et al., 2013; Freynet et al., 2020; Geiger et al., 2023; Giles, 1970; Gluszek and Dovidio, 2010; Pantos and Perkins, 2013; Yueh and Pariyadath, 2023).

Stigma begins with distinguishing and labeling certain human difference (Link and Phelan, 2001). Speaking with an accent is a characteristic signaling foreignness, being non-native (Gluszek and Dovidio, 2010). When non-accent is assumed to be the norm and standard, having an accent is labeled as abnormal and deviant. Such human difference is then associated with negative attributions and perceptions (Link and Phelan, 2001). Non-native accents are perceived to be inferior, less desirable, and less pleasant to listen to (Cargile et al., 1994; Edwards, 1999; Giles, 1970; Lippi-Green, 1997). Speaking with accents is associated with negative evaluations such as being incompetent, less intelligent, lazy, uneducated, less loyal, and less attractive (Adank et al., 2013; Bresnahan et al., 2002; Dragojevic et al., 2013; Geiger et al., 2023; Gluszek and Dovidio, 2010; Lorenz et al., 2024; Pantos and Perkins, 2013).

Furthermore, the way one speaks can convey abundant social information (Giles and Johnson, 1987). Accent is a salient cue for social categorization. A non-native accent is a clear marker that a person is an outgroup member (Kozlowski, 2015). Stigma is enacted by differentiating us from them, that is to create separation and keep away from outgroup members (Link and Phelan, 2001). Indeed, “accent becomes a litmus test for exclusion, an excuse to turn away, to refuse to recognize the other” (Lippi-Green, 1997, p. 64). Victims of stigma are oftentimes excluded from access to social resources, are downward placed in social hierarchy, and are exposed to structural discrimination (Herek, 2009; Link and Phelan, 2001; Parker and Aggleton, 2003). Stigma on accent can result in behaviors of humiliation, rejection, ostracism, hate, and aggression (Dovchin, 2020; Piller, 2016). People with accents are more likely to suffer unfair treatments in employment or other important situations (Geiger et al., 2023; Gluszek and Dovidio, 2010).

### 1.2 Accent stigma on international students

During interpersonal interactions, speaking with an accent is very noticeable and hard to conceal. The visibility of having an accent makes it an easy target for stigmatization. In fact, people are aware of being stigmatized due to their non-native accents (Jaspal and Sitaridou, 2013). Targets of accent stigma oftentimes feel being devalued and vulnerable. They suffer from psychological distress such as fear, shame, anxiety, isolation, and lack of belonging (Dovchin, 2020; Gluszek and Dovidio, 2010). Victims of accent stigma also experience identity threats, reduced self-efficacy, decreased self-esteem, social withdrawal, and so on (Dovchin, 2020; Freynet et al., 2020; Jaspal and Sitaridou, 2013).

International student is one such group frequently encountered with accent stigma (Dovchin, 2020; Park et al., 2022; Zhu and Bresnahan, 2021). Studying and living in a foreign country other than one’s own can be a stressful experience, as one has to make a variety of psychological and sociocultural adjustments (Searle and Ward, 1990; Ward and Kennedy, 1994). While international students are trying to navigate their way through a new culture, concern with one’s accent can present considerable obstacles on the road. International students who perceive stigma on their non-native accents can feel alienated and experience a sense of strangeness. They perceive hostility from the host culture and feel unwelcome and unsafe (Dovchin, 2020). International students often receive negative evaluations and unfair treatments due to the way they speak. They may be the targets of discriminatory acts ranging from ridiculing and mockery, to rejection and exclusion, and to hatred and aggression (Dovchin, 2020; Gluszek and Dovidio, 2010; McDonough et al., 2022). As a result, international students who suffer from accent stigma may limit their contact with the locals and withdraw from participation in the host culture, which can further lead to psychological problems and poor adjustment outcomes.

### 1.3 Consequences on self-efficacy and acculturation

The experience of accent stigma can decrease international students’ levels of self-efficacy. Self-efficacy is defined as “beliefs in one’s abilities to organize and execute the courses of action required to produce given attainments” (Bandura, 1997, p. 3). Self-efficacy is belief in one’s capability to take effective actions to meet task demands and obtain desirable outcomes (Bandura, 1995, 1997; Wood and Bandura, 1989). In the context of intercultural adjustment, self-efficacy is sojourners’ belief that they are able to manage intercultural challenges and function well in the host culture (Black and Gregersen, 1991; Fan and Mak, 1998; Hua et al., 2020; Li and Gasser, 2005). International students who perceive high self-efficacy have confidence in their ability to effectively cope with difficulties and perform well in another culture. Unfortunately, when international students are targeted for accent stigma, they can experience decreased confidence and self-esteem, a loss of the sense of control, and feel decapacitated (Dovchin, 2020; Jaspal and Sitaridou, 2013). Accent stigma can reduce international students’ self-efficacy, leaving them feel powerless.

Self-efficacy plays a crucial role in intercultural adjustment (Bhaskar-Shrinivas et al., 2005; Gebregergis et al., 2020; Hua et al., 2020; de Saissy, 2009; Li and Gasser, 2005; Mesidor and Sly, 2016; Rujiprak, 2016). International students with low self-efficacy may give up easily when confronted with setbacks and obstacles. They are less motivated to approach intercultural challenges and do not actively participate in the host culture. In other words, self-efficacy affects the coping strategy of international students in response to a new environment, which relates to the concept of acculturation (Berry, 1980). Acculturation refers to the process of psychological and behavioral changes that take place after people have contact with a new culture (Berry, 1997, 2005). Depending on people’s orientation toward maintaining one’s heritage culture and their orientation toward participating in the host culture, there are four acculturation strategies (Berry, 2005): assimilation (i.e., dismiss heritage culture and embrace host culture), separation (i.e., maintain heritage culture and avoid host culture), marginalization (i.e., dismiss heritage culture and avoid host culture), and integration (i.e., maintain heritage culture and participate in host culture). Compared to other acculturative strategies, integration leads to the best psychological and sociocultural adjustment outcomes (Berry, 2005). Research showed that adopting an integration strategy is associated with less acculturative stress, lower depression, higher self-esteem, higher life and education satisfaction (Chen et al., 2008; Coatsworth et al., 2005; David et al., 2009; Kunst, 2021; Lefringhausen and Marshall, 2016; Nguyen and Benet-Martínez, 2013).

Based on the above discussion, the current paper intends to better understand international students’ experience of accent stigma and how it has impacts on their self-efficacy and integration into a new culture. Two research questions were proposed:

Research Question 1 (RQ1): How is accent stigma perceived by international students during interpersonal interactions?

Research Question 2 (RQ2): What are the negative impacts of perceived accent stigma on international students’ intercultural adjustment?

Specifically, this paper presents two studies. Study 1 probed how international students perceived accent stigma in their everyday interactions, and Study 2 investigated the effects of perceived accent stigma on perceived discrimination, perceived hate, fear, self-efficacy, integration, and their interrelationships. The current paper attempts to shed light on the negative consequences of perceived accent stigma and provide implications for intercultural adjustments among international students.

## 2 Study 1

The purpose of Study 1 was to understand how international students experience accent stigma in their day-to-day life. In-depth interviews were conducted with international students to gain understandings of accent stigma from a perceiver’s perspective. Then, thematic analysis was performed to derive themes that describe how accent stigma is enacted and perceived during interpersonal interactions.

### 2.1 Method

#### 2.1.1 Participants

A total of 15 international undergraduate students at a midwestern U.S. university participated in in-depth interviews. The researcher stopped recruiting more participants when no more new information emerged from interviews and the data were presumably saturated. Participants had an average age of 21.6 years old and 60% of the participants were female. Eight participants came from mainland China, four from South Korea, two from India, and one from Pakistan. Participants volunteered to take part in this study and earned research credits upon completion.

#### 2.1.2 Procedure and design

All study procedures and materials gained approval from the Institutional Review Board at the university. The interviews were semi-structured and lasted for about 40 min each. Interviews were audio recorded for notetaking purposes. Upon the arrival of each participant, the researcher greeted him/her and obtained informed consent.

The researcher followed a protocol when having conversations with each participant, but the interview questions were adjusted based on the conversation flow. Each participant was first asked about their experience living in a foreign country, especially in terms of language and communication. Then, participants were asked to recall specific incidents when they had either observed or had been themselves targeted by accent stigma. During the recall, participants were prompted to think of what the stigmatizers said and did that made them perceive stigma toward their non-native accent. Participants were also asked about how they interpreted the meaning of these verbal messages and nonverbal behaviors.

Toward the end of each interview, demographic information was collected. Then, a buffer message was communicated to each participant to mitigate potential negative impacts he/she might have experienced during the interview. Lastly, the researcher thanked the participant and granted compensation.

#### 2.1.3 Data analysis

Reflexive thematic analysis (Braun and Clarke, 2006) was conducted on the transcripts of the interviews to identify and analyze the patterns of meaning. This approach to thematic analysis emphasizes “the researcher’s interpretive analysis of the data conducted at the intersection of: (1) the dataset; (2) the theoretical assumptions of the analysis, and; (3) the analytical skills/resources of the researcher” (Byrne, 2022, p. 1393).

The researcher followed a six-phase process proposed by Braun and Clarke (2012): (1) Familiarization with the data—the researcher read each transcript multiple times while highlighting interesting texts and taking notes of initial thoughts. (2) Generating initial codes—the researcher produced descriptive or interpretative labels for data items that might be useful in addressing the research question. (3) Generating themes—the researcher reviewed and analyzed the pattern of the initial codes and combined codes with shared meanings to form potential themes. (4) Reviewing potential themes—the researcher reviewed and modified the candidate themes to ensure that the codes inform each theme form a coherent pattern and the themes provide meaningful interpretation of the data in relation to the research question. (5) Defining and naming theme—the researcher distinguished each theme and created narratives that are consistent with the data and informative of the research question. (6) Producing the report—the researcher identified data extracts that provide compelling accounts of the meanings in each theme and reported themes in a logical manner.

### 2.2 Results

Through thematic analysis, four themes were developed and they were labeled: (1) verbal disapproval, (2) verbal avoidance, (3) nonverbal disapproval, and (4) nonverbal avoidance.

The first theme was verbal expressions of disapproval. This included verbal messages that were judging and meant to express that a condition (i.e., speaking with non-native accents) was less than satisfying. Such expression was also accompanied with a connotation that a condition was abnormal and deviant, and the person who had that condition was thought to be inferior, less competent, defective, or even less than a person. These expressions reflected stereotypes and prejudice. Here are some examples:

*I had no idea you would be mumbling like that.*

*You sound really funny.*

*Can you even talk like normally?*

*Is your English good enough to be my TA (teaching assistant)?*

*You cannot present our paper because you have an accent.*

The second theme was verbal avoidance. This theme described the lack of verbal communication, which represented the avoidance of or withdrawal from social interactions. The absence of expected communication reflected social distancing. As participants recalled, stigmatizers would not talk as much, became silent, or would not ask questions back. These examples showed that stigmatizers expressed verbal avoidance through reduced amount of verbal exchange, reduced conversation engagement, and a lack of communication intention.

The third theme was nonverbal expressions of disapproval. Oftentimes, stigmatizers expressed their feelings through more implicit and subtle channels. Instead of verbally articulate their disapproval of a condition, stigmatizers often use nonverbal cues as a silent protest. This is because stigmatizers understood that stigmatizing was socially undesirable, and explicit stigmatizing may face social sanctioning. For example, participants mentioned that stigmatizers would give weird looks, roll their eyes, make sounds like “Ugh!” or deeply sigh.

The last theme was nonverbal avoidance. This theme included nonverbal behaviors that stigmatizers demonstrate to socially distance or avoid the target of stigma. For instance, closed gestures, minimal physical contact, indifferent facial expression, and brief eye contact are all examples mentioned by participants. These nonverbal cues were subtle, but powerful. As participants interpreted, stigmatizers used nonverbal avoidance to declare differentiation and separation.

### 2.3 Discussion

Study 1 provided insights into how international students perceive accent stigma to be enacted and communicated during interpersonal encounters. As a response to RQ1, the in-depth interviews uncovered observable behavioral markers that signal the enactment of accent stigma. International students were made aware of the stigma targeted at their non-native accents when they perceived cues of verbal disapproval, verbal avoidance, nonverbal disapproval, and nonverbal avoidance.

These findings also inspire in more general ways as to how stigma can be manifested in interpersonal interactions. Stigma can be communicated through two channels: verbal or nonverbal. Verbal behaviors are associated with the manner in which we communicate with words, such as the amount of verbal exchange, turn-taking patterns, etc. Nonverbal behaviors are associated with the manner in which we communicate with nonlinguistic or paralinguistic cues, such as facial expressions, gestures, eye contact, voice, etc. Moreover, stigma can be enacted through two behavioral categories: to approach (in this case, to disapprove) or to avoid. This coincides with the two systems that regulate behaviors: behavioral activation system that motivates behaviors to approach rewards or positive outcomes, and behavioral inhibition system that inhibits behaviors to avoid punishments, threats, or negative outcomes (Gray, 1990). In the context of stigma, by expressing disapproval, stigmatizers seek for a higher status and a sense of superiority, reinforcing that they are the one in power and in control to decide what is abnormal, inferior, and undesirable. Also, stigmatizers use avoidance behaviors to demonstrate differentiation, separation and distancing, so as to protect oneself from the danger and threat posed by the target. These two behavioral systems, coupled with verbal and nonverbal channels, are able to capture the key aspects of stigma enactment. Stigma is directed toward a target with an undesirable condition (in this case, international students with non-native accents) through verbal or nonverbal behaviors of disapproval or avoidance.

As discussed earlier, perceived accent stigma can result in a range of negative consequences such as psychological distress and sociocultural maladjustments among international students (Dovchin, 2020; Gluszek and Dovidio, 2010). In order to answer RQ2, the next study further investigates how perceived accent stigma can lead to detrimental effects during international students’ intercultural adaptation.

## 3 Study 2

The purpose of Study 2 was to understand the negative impacts of perceived accent stigma on international students. Specifically, a path model was constructed based on survey data to demonstrate how perceived accent stigma and the associated negative perceptions can decrease international students’ self-efficacy and lead to poor integration into the host culture. Study 2 intends to provide implications for the adjustment experience of international students.

### 3.1 Method

#### 3.1.1 Participants

Participants were 132 international students at a midwestern U.S. university whose mother language is not English. Participants ranged from 18 to 40 years in age, with an average of 25.52 years (*SD* = 5.60). A little more than half of the participants were female (53.5%). Participants came from 34 different countries or districts around the world, with the most from mainland China (30.5%), followed by India (14.1%), South Korea (7.8%), Taiwan (5.5%), Malaysia (3.9%), Brazil (3.1%), Vietnam (2.4%), and Japan (2.3%). Participants’ duration of stay in the U.S. ranged from 0.25 to 15 years (*M* = 3.24, *SD* = 2.69). Participants came from more than 50 diverse majors in college. Among them, 40.6% were undergraduate students (14.7% freshmen, 5.5% sophomore, 14.1% junior, and 6.3% senior) and 59.4% were graduate students (16.4% master’s and 43% doctoral).

#### 3.1.2 Procedure and design

This study included an online survey on *Qualtrics*. The survey link was distributed with an invitation e-mail sent through the university’s registrar’s office to all international students at the university. Participants volunteered to complete the survey. The survey asked about participants’ experience of speaking English as a second language and various aspects of intercultural adaptation. All study procedures and materials were approved by the Institutional Review Board at the university.

At the very beginning of the survey, informed consent was obtained from all participants. A question filtered out international students who speak English as their first language (e.g., Canadian international students). Eligible participants then indicated their perceptions of stigma against them speaking accented English. Participants also answered questions about their perceived discrimination, perceived hate, fear, self-efficacy, and integration. Next, demographic information including age, gender, mother country, duration of stay in the U.S., year and major in school were collected. Then, all participants read a buffer message that intended to mitigate any negative impacts the survey might have caused on them. Participants who needed further help on mental health were provided with resources to counseling services on campus. Finally, participants were directed to a separate page not linked to the survey to enter into a lottery to win a 20-dollar Amazon Gift Card.

#### 3.1.3 Measurements

Scale items were obtained and derived from previous research. Participants marked their responses on 7-point Likert scales from 1 (strongly disagree) to 7 (strongly agree). Confirmatory Factor Analysis (CFA; Hunter and Gerbing, 1982) assessed a one-factor solution for each scale before an average score was calculated to represent participants’ standing on a certain variable. All scales demonstrated acceptable validity and reliability. Each measurement scale was described in details below.

*Perceived Accent stigma* was consisted of four subscales—verbal disapproval, verbal avoidance, nonverbal disapproval, and nonverbal avoidance. *Verbal disapproval* was measured with five items (e.g., “People have said bad things about me because I speak with accents” and “I was told that I am not good enough because I have accents when speaking”). CFA showed acceptable model fit, χ ^2^(5) = 4.10, *p* = 0.54, CFI = 1.00, TLI = 1.00, RMSEA = 0.00, SRMR = 0.02. Cronbach’s α = 0.89. *Verbal avoidance* was measured with eight items (e.g., “People do not pay attention to what I say because of my broken English” and “I was excluded from conversations due to my struggles with English pronunciations”). CFA showed acceptable model fit, χ ^2^(20) = 15.89, *p* = 0.72, CFI = 1.00, TLI = 1.00, RMSEA = 0.00, SRMR = 0.01. Cronbach’s α = 0.96. *Nonverbal disapproval* was measured with five items (e.g., “People roll their eyes at me because I have foreign accents” and “People have frowned at me for my broken English”). CFA showed acceptable model fit, χ ^2^(5) = 3.57, *p* = 0.61, CFI = 1.00, TLI = 1.00, RMSEA = 0.00, SRMR = 0.02. Cronbach’s α = 0.87. *Nonverbal avoidance* was measured with six items (e.g., reverse coded “People are willing to be around me regardless of my accented English” and reverse coded “Nobody has walked away from me during interactions because I speak English with accents”). CFA showed acceptable model fit, χ ^2^(9) = 16.64, *p* = 0.06, CFI = 0.98, TLI = 0.97, RMSEA = 0.08, SRMR = 0.04. Cronbach’s α = 0.87. Since perceptions of accent stigma was induced by the four subscales, it was treated as a formative or composite (rather than reflective or latent) variable (Edwards and Bagozzi, 2000). Therefore, an index of perceived accent stigma was computed by averaging scores across the four subscales, with higher scores representing higher perceptions of accent stigma (*M* = 2.69, *SD* = 1.13).

*Perceived discrimination* was measured with four items from Sandhu and Asrabadi (1994). Example items are “I am treated differently in social situations” and “I am denied what I deserve.” CFA showed acceptable model fit, χ ^2^(2) = 0.68, *p* = 0.71, CFI = 1.00, TLI = 1.00, RMSEA = 0.00, SRMR = 0.01. Cronbach’s α = 0.86. Higher scores on this scale meant more perceived discrimination (*M* = 3.58, *SD* = 1.50).

*Perceived hate* was measured with four items Sandhu and Asrabadi (1994). Example items are “Others do not appreciate my cultural values” and “People show hatred toward me through actions.” CFA showed acceptable model fit, χ ^2^(2) = 2.94, *p* = 0.23, CFI = 1.00, TLI = 0.99, RMSEA = 0.06, SRMR = 0.02. Cronbach’s α = 0.89. Higher scores on this scale meant more perceived hate (*M* = 2.67, *SD* = 1.32).

*Fear* was measured with three items from Sandhu and Asrabadi (1994): “I fear for my personal safety because of my different cultural background,” “I feel insecure here,” and “I generally keep a low profile due to fear.” Cronbach’s α = 0.90. Higher scores on this scale meant more fear (*M* = 2.92, *SD* = 1.70).

*Self-efficacy* was measured with seven items from Khawaja et al. (2014) that focus on self-efficacy in an intercultural context. Example items are “I know where to get help when in trouble,” “I can manage my two worlds” and “I can take care of myself in a new place.” CFA showed acceptable model fit, χ ^2^(14) = 21.64, *p* = 0.09, CFI = 0.98, TLI = 0.97, RMSEA = 0.06, SRMR = 0.04. Cronbach’s α = 0.82. Higher scores on this scale meant higher intercultural self-efficacy (*M* = 5.52, *SD* = 0.94).

*Integration* was measured with six items from Khawaja et al. (2014). Example items are “I am okay with accepting both American and my own cultural values,” “I use some American ways to deal with my problems,” and “My cultural values help me to deal with difficulties in America.” CFA showed acceptable model fit, χ ^2^(9) = 13.81, *p* = 0.13, CFI = 0.98, TLI = 0.97, RMSEA = 0.06, SRMR = 0.04. Cronbach’s α = 0.85. Higher scores on this scale meant better integration into the American society (*M* = 5.09, *SD* = 1.10).

### 3.2 Results

**Figure 1:**
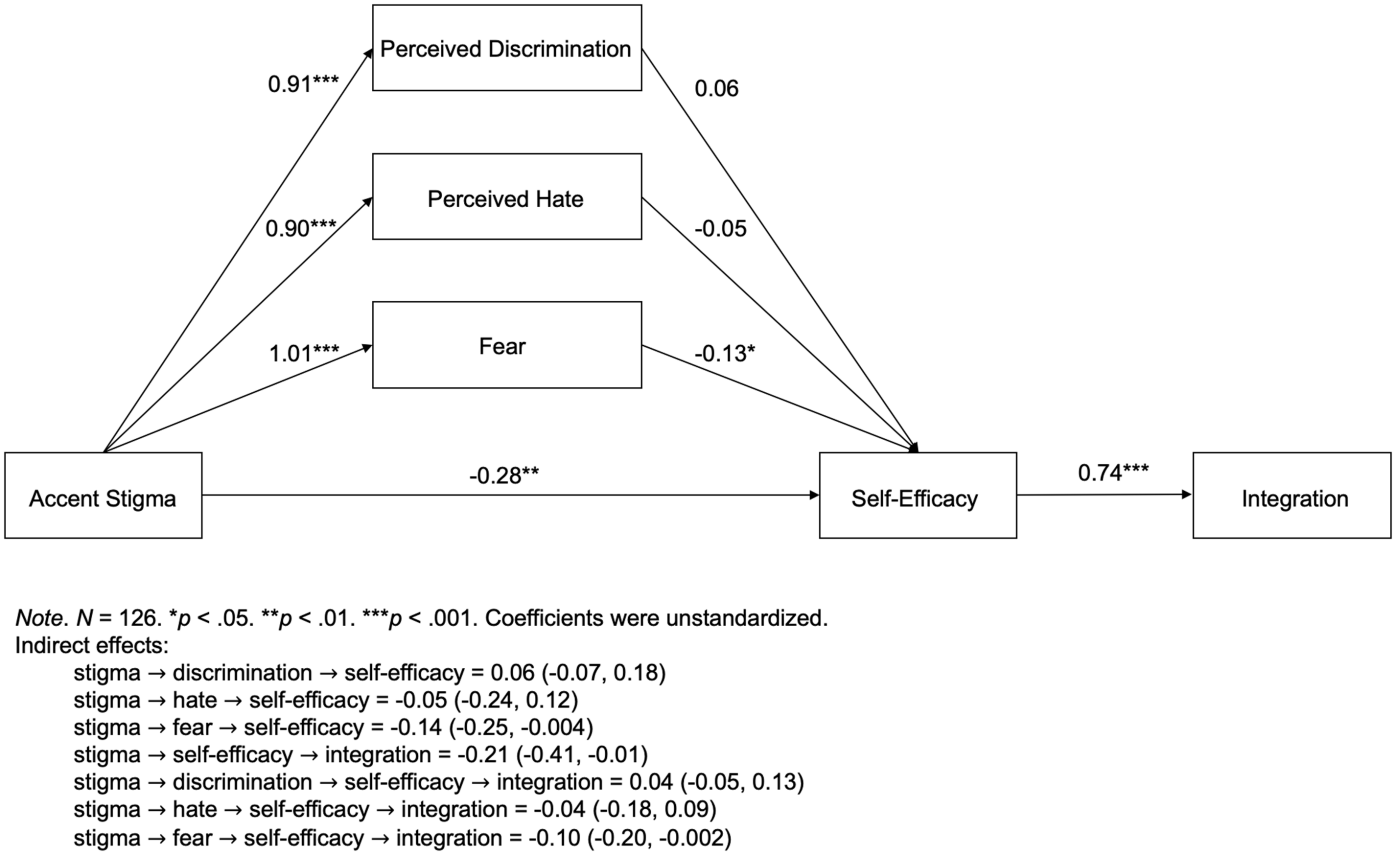
Path Model.

A path model (Figure 1) was assessed with *PROCESS* (*version 3.3* for *SPSS*). Unstandardized regression coefficients (*B*) were reported in this paper. Results showed that perceived accent stigma was significantly associated with more perceived discrimination (*B* = 0.91, *p* < 0.001), more perceived hate (*B* = 0.90, *p* < 0.001), and more fear (*B* = 1.01, *p* < 0.001). This suggested that international students who encountered accent stigma perceived more discrimination and hatred from the host culture and experienced more fear.

Holding perceived discrimination, perceived hate, and fear constant, perceived accent stigma had a significant direct effect on lower self-efficacy (*B* = −0.28, *p* < 0.01), which in turn, was significantly associated with poorer integration (*B* = 0.74, *p* < 0.001). The indirect effect of perceived accent stigma on integration mediated by self-efficacy was −0.21 with a 95% bootstrapped confidence interval being (−0.41, −0.01). Additionally, there was a significant indirect effect of perceived accent stigma on integration through fear and then through self-efficacy. The serial mediation effect was −0.10 with a 95% bootstrapped confidence interval being (−0.20, −0.002).

These results showed that international students’ perceived accent stigma both directly and indirectly decreased their self-efficacy—the belief in one’s capability to successfully perform a task or to produce an intended outcome. Low self-efficacy further resulted in poor integration into the host culture. In other words, perceived accent stigma harmed international students’ confidence in dealing with intercultural challenges and impeded them from surviving and thriving in a new culture.

### 3.3 Discussion

In answering RQ2, Study 2 investigated how perceived accent stigma can have negative impacts on international students’ intercultural experience. International students who were targeted for accent stigma perceived more hostility and hatred from the host culture and felt unsafe and unwelcome in a foreign land. Bearing accent stigma also decapacitated international students, leaving them feel unconfident and powerless when faced with intercultural situations. Ultimately, accent stigma can result in maladjustments and poor integration into the host culture.

This paper provided better understanding of accent stigma from the perspective of the perceiver (i.e., international students). Consistent with previous research findings, accent stigma can lead to psychological distress such as fear and anxiety, decreased self-esteem and self-efficacy, discrimination, and social withdrawal (Dovchin, 2020; Freynet et al., 2020; Gluszek and Dovidio, 2010; McDonough et al., 2022). This paper further contributed to existing literature by disentangling the interrelationships among the consequences of accent stigma. Specifically, this paper systematically tested self-efficacy as a mechanism through which perceived accent stigma led to poor integration into the host culture. These findings highlighted the crucial role self-efficacy plays during intercultural adjustment.

The path model showed that self-efficacy mediated the impacts of perceived accent stigma on integration. In other words, one mechanism that accent stigma negatively affected international students was by hurting their confidence and efficacious beliefs. This echoed with research suggesting that perceived rather than actual language ability was more important to intercultural adjustment outcomes (Swagler and Ellis, 2003; Tskhay and Nguyen, 2011; Yeh and Inose, 2003). As Bandura (2005) argued, it is what people believe that forms the basis of human motivation. One’s perceived ability to achieve desirable outcomes is an important source of human agency (Bandura, 1997; Wood and Bandura, 1989). International students suffering accent stigma would develop negative self-views and lose confidence, which in turn, would reduce their motivation to proactively cope with intercultural challenges and to get involved in the host culture.

The mediating role of self-efficacy also had important practical implications for intervention programs aimed to help and support international students during their stressful transitional period in a new culture. Orientation programs and counseling services may put emphasis on boosting international students’ self-efficacy, in order to promote favorable intercultural adjustment outcomes. Professionals may accomplish this by guiding international students to discover their strengths and remind them of the areas they are confident in (Lin and Betz, 2009; Lin and Pedersen, 2007). Interventions may revolve fostering personal resilience, celebrating accomplishments, role modeling, providing social support, and so on (Lin and Betz, 2009; Lin and Pedersen, 2007). When international students feel efficacious, confident, and resourceful, they would be able to weather through difficult and challenging intercultural situations and see their intercultural experience as a rewarding adventure.

Admittedly, this study had some limitations. For instance, this study investigated accent stigma in general without distinguishing speakers’ countries of origin. This was based on evidence showing that the foreignness of a non-native accent is sufficient to activate general stereotypes about the speaker, without the need to correctly identity the accent (Milroy and McClenaghan, 1977). Nonetheless, one may still question this “one size fits all” approach (Schwartz et al., 2010; Rudmin, 2003). It is possible that differences might exist among international students with different countries of origin in terms of their intercultural experience. For example, the relationship between one’s home country and the host country may influence one’s perception of hatred and hostility from the host culture and affect one’s levels of anxiety and stress when one interacts with the host community, which can shape one’s intercultural adjustment and acculturation strategy. Therefore, future studies are encouraged to identify key variables that may explain variances among international students and to address issues unique and specific to a certain subset of international students.

## 4 Conclusion

Studying and living in a foreign country other than one’s own can be a challenging experience. One of the challenges for international students is presented by stigma on their non-native accent. International students may perceive accent stigma when disapproval and avoidance are communicated verbally or nonverbally during interpersonal interactions. Concern with one’s accent can result in a range of negative consequences such as perceived discrimination, perceived hate, and fear. International students who perceive accent stigma can also have decreased self-efficacy, which in turn, lead to poor integration into the host culture. In all, this paper provided better understanding of accent stigma from the perspective of the perceiver (i.e., international students), and highlighted the mediating role of self-efficacy during intercultural adjustment.

## Data Availability

The raw data supporting the conclusions of this article will be made available by the authors, without undue reservation.
